# Morphology control and optical properties of SiGe nanostructures grown on glass substrate

**DOI:** 10.1186/1556-276X-7-155

**Published:** 2012-02-27

**Authors:** Hsu-Kai Chang, Si-Chen Lee

**Affiliations:** 1 Graduate Institute of Electronics Engineering, National Taiwan University, Taipei, 10617, Taiwan; 2 Department of Electrical Engineering, National Taiwan University, Taipei, 10617, Taiwan

**Keywords:** SiGe, reflectance, nanowire, core-shell, transformation

## Abstract

With the rapid progress of nanotechnology, nanostructures with different morphologies have been realized, which may be very promising to enhance the performance of semiconductor devices. In this study, SiGe nanostructures with several kinds of configurations have been synthesized through a chemical vapor deposition process. By controlling growth conditions, different SiGe nanostructures can be easily tuned. Structures and compositions of the nanostructures were determined by scanning electron microscopy, transmission electron microscopy, and X-ray diffraction. The optical properties of various SiGe nanostructures revealed some dependence with their morphologies, which may be suitable for solar cell applications. The control of the SiGe morphology on nanoscale provides a convenient route to produce diverse SiGe nanostructures and creates new opportunities to realize the integration of future devices.

## Introduction

Semiconductor nanostructures, such as nanowires, nanotubes, and nanoflowers, have been extensively studied as building blocks for emerging devices [1-3]. Recently, a substantial interest has focused on the synthesis of one-dimensional nanostructures because they are expected to play a critical role as interconnects or functional units in fabricating promising nanodevices [4,5]. Among various kinds of nanomaterials, SiGe-based nanostructures are of great importance to study because they incorporate desirable characteristics of Si and Ge. With a low power consumption nature, SiGe-based electronic devices may achieve better performance than Si-based ones. Besides, SiGe provides additional flexibility through bandgap engineering, and it is also compatible with standard semiconductor processing.

However, due to different thermodynamics and kinetics of SiH_4 _and GeH_4 _[6,7], it is very challenging to achieve controlled growth of SiGe nanowires and their heterostructures by vapor-liquid-solid (VLS) method [8]. To explore the unique properties of SiGe nanostructures, detailed understanding of their characteristics in different growth conditions is required. Moreover, for the fabrication of versatile nanoscale devices, developing morphology-controlled growth of SiGe nanostructures is also an important issue.

The present work reports the fabrication of SiGe nanostructures using a chemical vapor deposition (CVD) method under different growth conditions. Our motivation is to find the morphology dependence on the nanostructures' preparation parameters. The optical properties of the synthesized SiGe nanostructures were also investigated.

## Experimental procedure

The experiments were carried out in a hot-wall thermal chemical vapor deposition system using GeH_4 _(10% premixed in N_2_) and SiH_4 _(10% premixed in N_2_) as the precursor gases. Glass substrates (Corning 1737F, Corning Inc., Corning, NY, USA) were first cleaned in piranha solution (3:1 (*v*/*v*) H_2_SO_4_/H_2_O_2_) and sonicated in DI water. Subsequently, poly-L-lysine solution was dripped on several pieces of cleaned glass substrates. After that, commercially available Au nanoparticles were deposited on these poly-L-lysine functionalized glass substrates. The substrates were loaded into the deposition chamber after removing poly-L-lysine. During the heating period, the reaction chamber was flushed with N_2 _and pumped out with a mechanical pump. The reaction temperature was varied from 405°C to 475°C, which is well controlled by a computer. The total pressure in the reaction chamber was fixed at 30 Torr. The flow rate of SiH_4 _was maintained at a constant value, but the flow rate of GeH_4 _was set to 24 or 40 sccm.

The structure and morphology of the as-grown samples were examined by field emission scanning electron microscopy (Gemini LEO 1530, Carl Zeiss Microscopy, Carl-Zeiss-Straße, Oberkochen, Germany) and by high-resolution transmission electron microscopy (300 kV Philips Tecnai F30, FEI Co., Hillsboro, OR, USA) equipped with an energy dispersive X-ray (EDX) detector. The phases and crystal orientation analysis of the synthesized nanowires were identified by X-ray diffraction (XRD; Cu Kα radiation, X'pert, PANalytical B.V., Almelo, The Netherlands) with a Bragg angle ranging from 20° to 80°. In order to explore the optical properties of different SiGe nanostructures, the reflectance spectra were also measured using a UV-1650PC spectrometer (Shimadzu Corporation, Nakagyo-ku, Kyoto, Japan) for a wavelength range from 350 to 1,000 nm.

## Results and discussion

### SiGe nanorod growth

**Figure 1:**
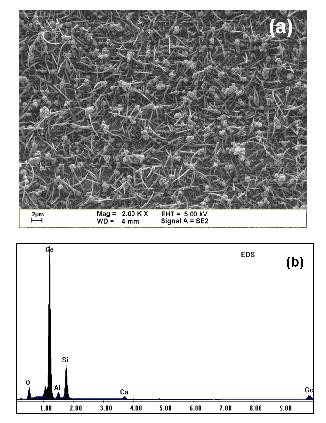
**SEM image and EDX data**. (**a**) SEM image of nanorods grown at 405°C and (**b**) corresponding EDX results.

**Figure 2:**
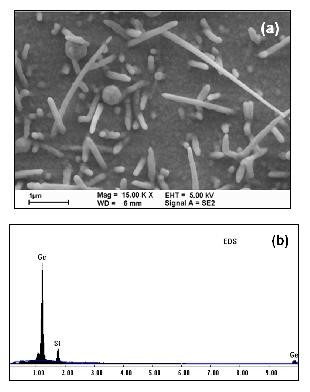
**SEM image and EDX data**. (**a**) SEM image of nanorods grown at 438°C and (**b**) corresponding EDX results.

Due to the relatively lower eutectic temperature of Au/Si and Au/Ge alloys (approximately 363°C for Au/Si and 361°C for Au/Ge), gold as catalyst is particularly suitable for one-dimensional SiGe nanostructure growth. In order to achieve a higher growth rate, the experiments were started at 405°C. Figure 1a shows a scanning electron microscopy (SEM) image of SiGe nanorods grown at 405°C. The as-grown samples are randomly oriented with lengths shorter than 5 μm. The EDX analysis shows that the element Ge in SiGe nanorods has a higher concentration compared to the element Si, as demonstrated in Figure 1b. The signals of aluminium, oxygen, and calcium in this EDX data are mainly from the glass substrate. When the growth temperature is heated to 438°C, however, the yields of the SiGe nanorods are totally decreased, as can be seen from Figure 2a. Besides, the lengths of the nanorods are shorter than 2 μm even though the growth time is the same with the sample grown at 405°C. From EDX data, as shown in Figure 2b, we found that the Ge concentration at this temperature is higher than Si as well. If we move the growth temperature to 462°C, the yields will be highly improved, but some instability on the surfaces of the SiGe nanostructures will occur. This instability results in a bead-like structure, which means wavy sidewalls are along individual SiGe nanowires. This phenomenon has been observed before in Si whisker growth via the VLS method and was attributed to a self-oscillation mechanism [9].

### SiGe/Ge core-shell nanowire growth

In order to find the condition for eliminating the bead-like structure, we tried to choose a much higher temperature for the growth, wherein we wish more germane decomposition to cover the wavy sidewall. The reason may be ascribed to the larger activation energy for the decomposition of silane than that of germane [10]. Some group has also observed the increase in Ge deposition [11] at a higher temperature. In addition, several theoretical studies also indicated that a core-shell structure could be more stable than a homogenous structure [12,13]. However, at a higher temperature such as 475°C, there might be some oxide deposition on the sidewalls of our nanowires due to the residue oxygen in the reaction chamber. To confirm whether the oxygen will participate in the deposition, we performed EDX analysis to the samples grown at 475°C, and no oxygen content was detected.

**Figure 3:**
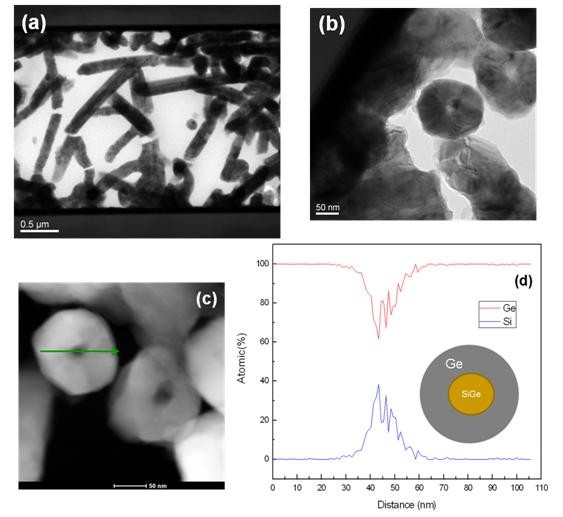
**TEM image and EDX data**. (**a**) TEM image of nanowires grown at 475°C, (**b**) cross-sectional TEM images, (**c**) HAADF TEM images of a core-shell structure, and (**d**) EDX data from a core-shell structure.

A detailed microstructure information and morphology variation of the as-grown samples were further characterized by transmission electron microscopy (TEM). Figure 3a demonstrates the TEM image of the Ge shells on top of the SiGe core nanowires. Figure 3c shows the high-angle annular dark field (HAADF) TEM image in a cross-section view. The core-shell nature can be clearly differentiated from the black and white contrast. For a detailed composition of a single core-shell nanowire, see Figure 3d. Since we did not find the Au catalyst on top of the nanowire, the growth mechanism for this core-shell structure may be through the vapor-solid (VS) method [14] or a combination of the VLS and VS methods. It is known that when VLS growth has stopped, direct CVD growth may take over on the Au-covered sidewalls of the SiGe nanowires (NWs). This may lead to the deposition of Ge on the VLS-grown nanowires, which results in an increase of the nanowire diameter [15].

**Figure 4:**
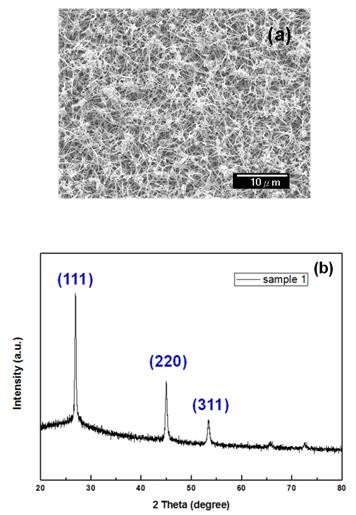
**SEM image and XRD data**. (**a**) SEM image of nanowires grown at 475°C and (**b**) corresponding XRD patterns.

To further explore the crystal quality of SiGe/Ge NWs, we also performed the XRD measurement. Figure 4a is the typical SEM image of as-grown SiGe/Ge NWs, which we used for XRD studies. Figure 4b shows the XRD patterns of the prepared core-shell nanostructures deposited on a glass substrate. The diffraction peaks corresponding to the (111), (220), and (311) planes of SiGe were observed. The intensity of the diffraction peak at 2*θ *= 28° is much stronger than the others, showing that the predominant growth direction of the core-shell NWs is mainly along [111].

### Optical properties of SiGe nanostructures

**Figure 5:**
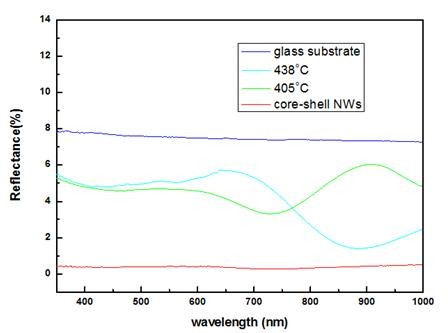
**Reflectance spectra**. Spectral reflectance of SiGe nanostructures measured with light incidents along the normal direction to the surface.

Nanowires may possess good antireflection properties for solar cell applications because the subwavelength scale of nanowire arrays will strongly scatter incident light and has a graded refractive index, which may enhance the incidence of light in usable wavelength range. Effective medium models predict this suggestion [16]. To find the antireflection performance on our samples, we also conduct the reflectance measurement on SiGe nanostructures grown at different temperatures. The reflection spectra were illustrated in Figure 5. Samples with a dark surface usually demonstrate reduced reflection due to enhanced absorption from the surface, but nanowires with core-shell structures may get lower reflection even though the sample has a light surface. It has been shown that core-shell nanowires exhibit good antireflection properties due to enhanced light trapping in these structures [17]. The reflectance of the sample with core-shell structures has been highly suppressed to less than 1% in the whole measured range. The nanorods grown at 405°C and 438°C have roughly the same reflectance from 350 to 450 nm. For reflection above 600 nm, both 405°C and 438°C samples start to exhibit interference effects. Since there are lower yields at 438°C than at 405°C, the sample grown at 438°C may have more thin film coating in the region where no nanorods grow. Due to the interference effects of the thin film coating, the sample grown at 438°C will show a local minimum in the reflectance curves. Therefore, it has lower reflection above 800 nm compared with the sample grown at 405°C. All the samples exhibit lower reflectance than their substrate, which means that the SiGe nanostructures have higher absorption.

## Conclusions

In summary, we have grown SiGe nanostructures under different growth conditions via a simple CVD method. Temperature-dependent morphology changes and their optical properties have been further discussed. The identification of different growth conditions for SiGe nanostructures has great potential for preparing diverse nanostructures, which may be suitable for multifunctional device applications.

## Abbreviations

CVD: chemical vapor deposition; NW: nanowires; SEM: scanning electron microscopy; TEM: transmission electron microscopy; XRD: X-ray diffraction.

## Competing interests

The authors declare that they have no competing interests.

## Authors' contributions

HKC participated in the realization of the project, carried out the experiments, and wrote the paper. SCL supervised the whole project, the experiments, and the interpretation. All authors read and approved the final manuscript.
